# A VIN3-like Protein OsVIL1 Is Involved in Grain Yield and Biomass in Rice

**DOI:** 10.3390/plants11010083

**Published:** 2021-12-28

**Authors:** Jinmi Yoon, Hee-Joong Jeong, Gibeom Baek, Jungil Yang, Xin Peng, Win Tun, Sun-Tae Kim, Gynheung An, Lae-Hyeon Cho

**Affiliations:** 1 Department of Plant Bioscience, Life and Industry Convergence Research Institute, Pusan National University, Miryang 50463, Korea; jmjh0120@khu.ac.kr (J.Y.); bakki96@pusan.ac.kr (G.B.); stkim71@pusan.ac.kr (S.-T.K.); 2 Crop Biotech Institute and Graduate School of Biotechnology, Kyung Hee University, Yongin 17104, Korea; hjjung@postech.ac.kr (H.-J.J.); rnlwo@postech.ac.kr (J.Y.); xinpenglyl@163.com (X.P.); wintun@khu.ac.kr (W.T.); 3 Institution of Genomics and Bioinformatics, South China Agricultural University, Guangzhou 510642, China

**Keywords:** rice, grain yield, biomass, histone modification, *OsVIL1*, *OsCKX2*

## Abstract

In chromatin remodeling, the post-translational modification of histone proteins is mediated by multimeric protein complexes. VERNALIZATION INSENSITIVE3 (VIN3) forms a complex with Polycomb Repressive Complex 2 (PRC2), which mediates the trimethylation of H3K27 to repress target gene expression. In rice, four genes (OsVIL1-OsVIL4) encoding the VIN3-like proteins are expressed ubiquitously in various tissues. Null mutants of *osvil2* display pleiotropic phenotypes such as altered flowering time, floral organ defects, and reduced tiller size. In contrast, *osvil1* mutants did not show significant phenotypes except in fertilization compared with the wild type. However, transgenic plants overexpressing *OsVIL1* showed phenotypes of increased biomass and grain yield. Cross-sections of the basal region of elongating stems revealed that the increased biomass was mediated by inducing cell proliferation in the meristem. Chromatin immunoprecipitation assay indicated that OsVIL1 repressed expression of cytokinin oxidase/dehydrogenase gene (*OsCKX2*) by binding to the promoter and genic regions of *OsCKX2*. We also observed that OsVIL1 modified the levels of H3K27me3 in the *OsCKX2* chromatin. Because *OsCKX2* encodes an enzyme that degrades active cytokinin, we conclude that OsVIL1 functions in the regulation of endogenous active cytokinin levels, thereby increasing plant height and productivity.

## 1. Introduction

Rice is a staple food, and increasing productivity of this crop is an important agricultural goal. To this end, much research has been carried out on increasing rice grain yield. In rice, plant architecture is an important factor that determines productivity, which is well known to depend on meristem activity [1,2]. Cytokinins are important in regulating meristem activity and maintenance by increasing cell division [1,2,3,4,5]. *LONELY GUY* (*LOG*) encodes a cytokinin riboside 5′-monophosphate phosphoribohydrolase that functions during the final step in bioactive cytokinin synthesis [3]. *LOG* mutants exhibit defective maintenance of shoot meristems and a severe reduction in panicle size [3]. The most effective QTL for increasing grain yield, *GRAIN NUMBER 1a* (*Gn1a*), encodes cytokinin oxidase/dehydrogenase (OsCKX2), which degrades active cytokinin [6]. Decreased expression of *OsCKX2* causes cytokinin to accumulate in inflorescence meristems and leads to a large number of tillers and spikelets per plant [6,7]. Several factors that influence grain productivity and biomass are associated with *OsCKX2* regulation [6,7,8]. The zinc-finger transcription factor DROUGHT AND SALT TOLERANCE (DST) directly activates *OsCKX2* expression in the meristem [9]. The mutant allele DST^reg1^ causes down-regulation of *OsCKX2*, thus increasing cytokinin accumulation in the inflorescence meristem and increasing the number of branches and grains [9]. Also, *LARGER PANICLE* (*LP*), which encodes a Kelch repeat-containing F-box protein, improves the panicle architecture and grain yield in rice [10]. LP is an ER-localized protein and interacts with rice SKP-like proteins, suggesting that LP might be involved in ER-associated protein degradation. Mutation of the *LP* gene reduces the expression of *OsCKX2* in young panicles, causing cytokinin accumulation. Also, OsVIL2 represses the expression of *OsCKX2* by direct interaction with the promoter region of *OsCKX2* [7]. In addition, a chromatin interacting factor OsVIL2 increases panicle development and biomass by increasing active cytokinin levels.

Polycomb repressive complexes contain two major protein complexes, PRC1 and PRC2, in plants [11,12]. PRC2s are methyltransferases that introduce trimethylation of lysine 27 on histone H3 (H3K27me3) to repress down-stream target genes [13,14]. In Arabidopsis, the VERNALIZATION 2 (VRN2)-PRC2 complex has several components: VRN2, SWINGER (an E(Z) Histone Methyltransferase homolog), FERTILIZATION-INDEPENDENT ENDOSPERM (ESC homolog), and Musashi-1 (p55 homolog), which form the canonical part, while three plant-specific PHD (plant homeodomain) finger proteins (VRN5, VERNALIZATION INSENSITIVE3, and VEL1) create the accessory part [13,15,16]. Previous reports have indicated that VRN2-PRC2 plays important roles in the vernalization pathway by mediating the accumulation of H3K27me3 at the *FLOWERING LOCUS C* (*FLC*) locus after vernalization [13,17,18]. The VRN2 complex functions in the vernalization pathway associated with VIN3, VIL1/VRN5, and VIL2 /VERNALIZATION-LIKE 1 [15,19]. VIN3 and VIN3-like proteins consist of conserved domains of the PHD finger, the fibronectin type III domain (FNIII), and the VIN3 interacting domain (VID). The PHD finger recognizes and binds to histone proteins, the FNIII domain is involved in protein-protein interactions, and VID domain is responsible for interactions between other VIL proteins [7,20,21].

Although the vernalization response is not involved in controlling flowering time in rice, it is associated with four VIN3-like proteins: *Oryza sativa VIN3-like 1–4* (*OsVIL1–4*) [7,22]. OsVIL2 binds to *O. sativa* EMBRYONIC FLOWER 2b (OsEMF2b), a component of PRC2, and the PHD-PRC2 complex induces flowering by repressing *O. sativa LEAFY COTYLEDON 2 and FUSCA 3-LIKE 1* (*OsLFL1*) [23]. Moreover, OsVIL1 binds to OsEMF2b and induces flowering by repressing *OsLF* under short-day conditions but delays flowering by inducing *Grain number, plant height, and heading date 7* (*Ghd7*) expression under long-day conditions [24,25]. OsVIL2 also plays essential roles in regulating meristem activity and affects biomass, grain yield, tiller outgrowth, and spikelet development [7,26,27]. Although both OsVIL1 and OsVIL2 are understood to control flowering time, how OsVIL1 affects other developmental processes has not been studied to date. Here, we focused on how OsVIL1 functions differ from those of OsVIL2, which have already been studied. We showed that OsVIL1 increases grain yield and biomass through the direct regulation of *OsCKX2* expression, similarly to OsVIL2.

## 2. Results

### 2.1. Mutation of OsVIL1 Produces No Obvious Phenotypes in Spikelet Development and Tiller Outgrowth

**Figure 1:**
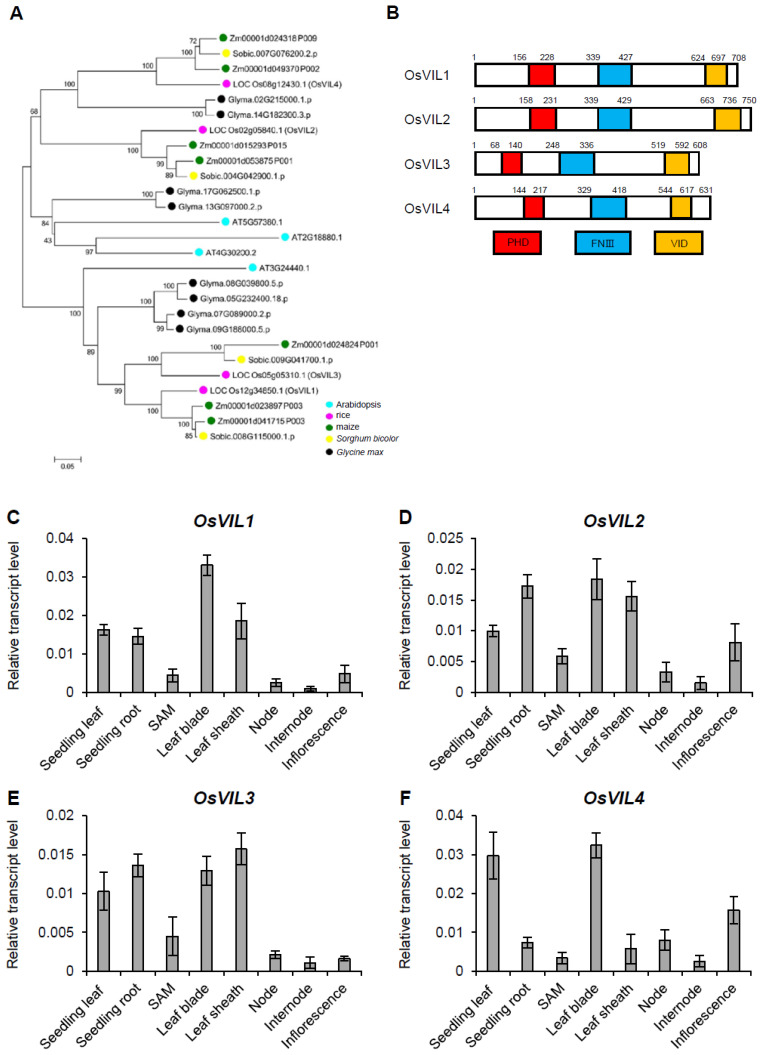
Comparative analysis of OsVIL genes. (**A**) Phylogenetic analysis of proteins OsVIL1–4 in rice, Arabidopsis, maize, *Sorghum bicolor*, and *Glycine max*. (**B**) Protein structures of OsVIL1–4. (**C**–**F**) Expression levels of *OsVIL1* (**C**), *OsVIL2* (**D**), *OsVIL3* (**E**), and *OsVIL4* (**F**) in various tissues. Rice *Actin1* was used as an internal control. The error bars indicate the standard deviations, *n* = 4.

The rice genome contains four VIN3-like genes: *OsVIL1*, *OsVIL2*, *OsVIL3*, and *OsVIL4* (Figure 1A). Interestingly, OsVIL genes extensively existed in higher plants, including lycophytes, gymnosperms, amborellales, eudicots, and monocots, which indicates their functional importance in these plants (Figure 1A). Similar to Arabidopsis VIN3-like proteins, OsVIL1–4 proteins contain PHD, FNIII, and VID domains (Figure 1B). The expression of the four OsVIL genes was broadly detected in various tissues of rice (Figure 1C–F and Supplementary Figure S1).

**Figure 2:**
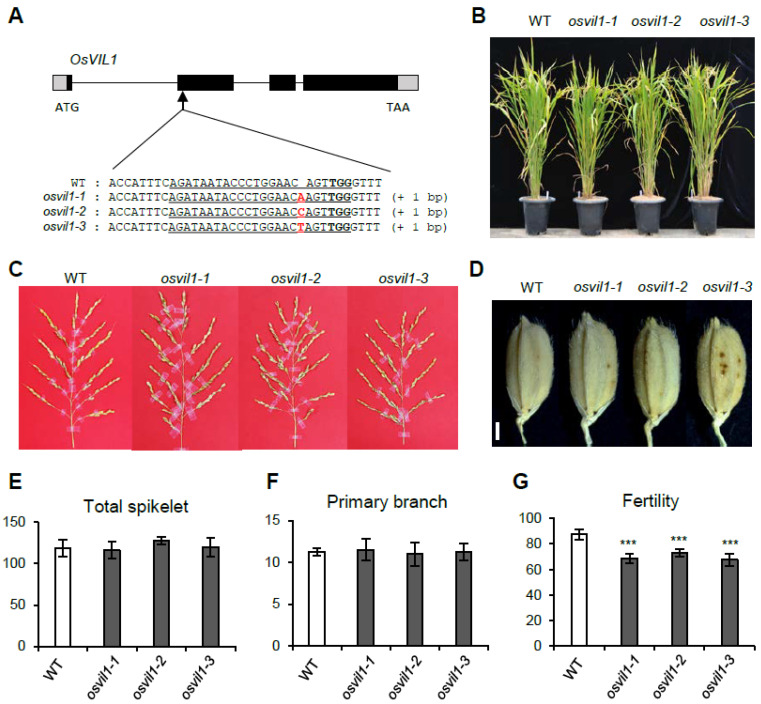
Phenotypes of the wild-type (WT) and *osvil1* null mutants. (**A**) Schematic structure of *OsVIL1* and mutation events obtained using the CRISPR/Cas9 method. In the gene structure, the black boxes indicate the four exons and the gray boxes indicate the 5′UTR and 3′UTR regions. The arrow indicates the mutation sites revealed using the CRISPR/Cas9 method. The target sequence is underlined with the protospacer adjacent motif shown in bold. Altered DNA sequences are indicated in red. (**B**) Phenotype of plant growth in WT and *osvil1* mutants at the seed-ripening stage under paddy field conditions. (**C**) Panicle phenotypes. Bar = 2 cm. (**D**) Seed phenotypes. Bar = 1 mm. (**E**) Total number of spikelets on one panicle. (**F**) Number of primary branches on the main panicle. (**G**) Seed fertility. The error bars indicate the standard deviations, *n* = Statistical significance is indicated by *** (*p* < 0.001).

To study the functions of OsVIL1, we generated *osvil1* null mutants using the CRISPR/Cas9 method. In the T_2_ generation, we found three independent lines containing 1-bp insertion that causes early termination of translation (Figure 2A). Compared with the wild type (WT), plant height, and tiller outgrowth were not different in these *osvil1* mutants (Figure 2B). The development of panicles and spikelets was also normal in the mutants (Figure 2C–F). Although there were no significant defects in floral organ development, fertility was slightly decreased in the mutants compared with the WT (Figure 2G). These results indicated that *OsVIL1* functions redundantly with OsVIL2 in most developmental processes except for a reduction in fertilization.

### 2.2. Overexpression of OsVIL1 Induces Increased Grain Yield and Biomass

**Figure 3:**
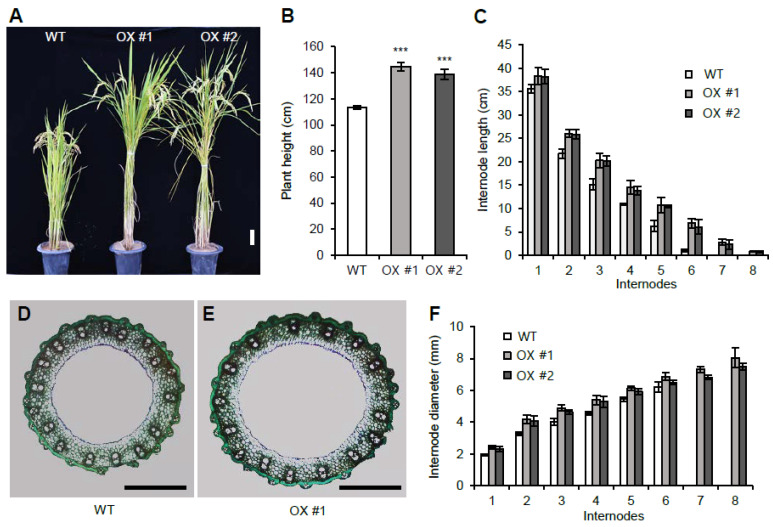
Internode development of wild-type (WT) and *OsVIL1* overexpression (OX) transgenic plants. (**A**) Phenotypes at about 152 days after sowing (DAS) in the paddy field. Bar = 10 cm. (**B**) Plants height at about 152 DAS. (**C**) Comparison of each internode length at about 152 DAS. (**D**,**E**) Cross sections of the main culm from the first internode of the WT and *OsVIL1* OX #1 transgenic line at about 112 DAS. Bars = 1 mm. (**F**) Comparison of the internode diameters at about 112 DAS. Error bars indicate the standard deviations, *n* = 4 or Statistical significance is indicated by *** (*p* < 0.001).

To determine the function of the other rice VIN3-like proteins, we generated *OsVIL1* OX transgenic plants (Figure 3A). Under natural paddy field conditions, the *OsVIL1* OX plants showed a >20% increase in height compared with the segregated WT (Figure 3B). When each internode length of the WT and *OsVIL1* OX plants was measured, we found that the length of each internode was increased in the OX plants compared with the WT (Figure 3C). In addition, two more elongated internodes were observed in the OX plants, indicating that increased plant height of the transgenics was due to increased internode number and length (Figure 3C). Cross-section of the stems showed that the diameter of the main culms was increased in *OsVIL1* OX plants when compared with the WT (Figure 3D–F).

**Figure 4:**
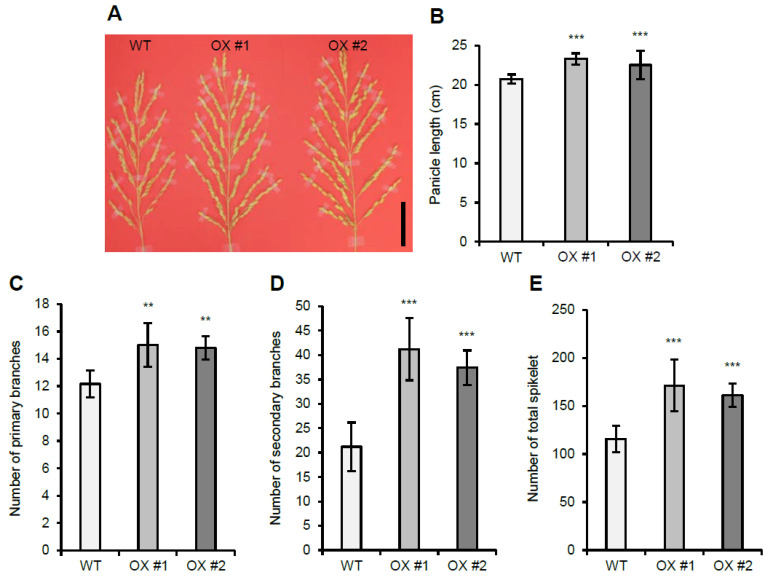
Panicle development in wild-type (WT) and *OsVIL1* overexpression (OX) plants. (**A**) Panicle phenotype of WT and *OsVIL1* OX transgenic lines. Bar = 5 cm. (**B**) Main panicle lengths. (**C**) Number of primary branches on the main panicle. (**D**) Number of secondary branches on the main panicle. (**E**) Total number of spikelets on the main panicle. The error bars indicate the standard deviations, *n* = Statistical significance is indicated by *** (*p* < 0.001), ** (*p* < 0.05).

Similar to the *OsVIL2* OX plants that exhibit increased grain yield, *OsVIL1* OX plants showed increased grain yield (Figure 4). *OsVIL1* OX lines had larger panicles (Figure 4A,B) and an increased number of primary (Figure 4C) and secondary branches (Figure 4D) compared with the WT. The increased number of primary and secondary branches caused an increase in the total number of spikelets per main panicle (Figure 4E).

### 2.3. Overexpression of OsVIL1 Affects Cell Numbers in Meristem Regions

**Figure 5:**
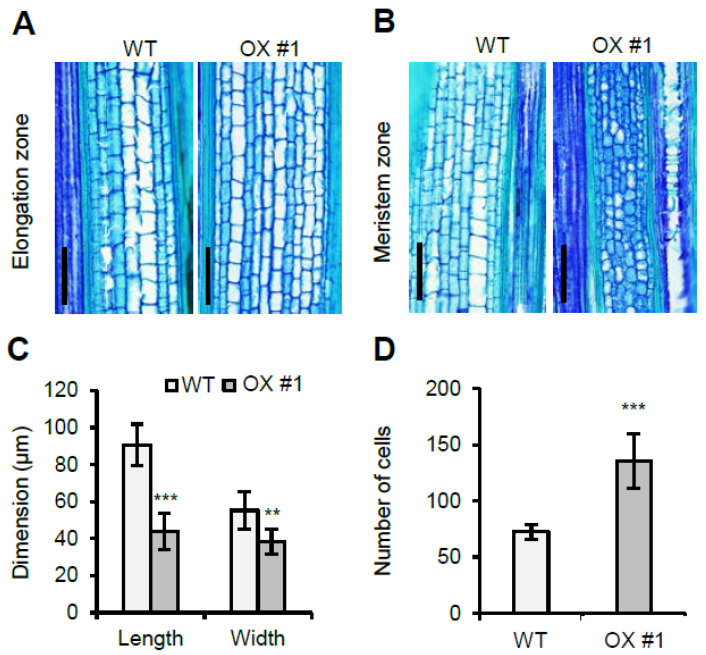
Longitudinal section of the first internode from wild-type (WT) and *OsVIL1* overexpression (OX) plants. (**A**) Longitudinal section analyses of the elongation zone from the first internodes in the WT and *OsVIL1* OX #1 line at about 149 DAS. Bars = 200 μm. (**B**) Longitudinal section analyses of the meristem zone from the first internodes at about 149 DAS. Bars = 200 μm. (**C**) Comparison of cell length and width in the upper 0.5 cm of the cell division region in the first internode of the WT and *OsVIL1* OX #1 line, *n* = 30 cells from three individual plants. (**D**) Comparison of cell numbers. Cells were counted in a sample 0.2-mm^2^ area in the meristem region of the internode. The error bars indicate the standard deviations, *n* = Statistical significance is indicated by *** (*p* < 0.001), ** (*p* < 0.05).

To investigate the differences between the WT and *OsVIL1* OX plants at the cellular level, we analyzed the size and number of the cells in the first internode at about 149 DAS. First, we checked the elongation region of the internode. The longitudinal section of the region showed that the cell number and size in the *OsVIL1* OX plants were not significantly increased compared with those of the WT (Figure 5A and Supplementary Figure S2). This observation suggested that increased plant height was not due to cell size. To evaluate whether the increase in plant height observed in the *OsVIL1* OX transgenic lines was due to increases in the cell number, the meristem region at the basal parts of the first internode were examined at the about 149 days after sowing (DAS) in the paddy field (Figure 5B). The cell size and number were analyzed in a 0.2-mm^2^ sample area in the division zone after longitudinal sections. The analysis indicated that cell size from the *OsVIL1* OX plants was decreased to approximately 52% and 30% in length and width, respectively (Figure 5C). In contrast, the cell number was significantly increased to about 180% in *OsVIL1* OX plants compared with the segregated WT (Figure 5D). These results suggested that *OsVIL1* promoted cell division in the meristem region of the stem.

### 2.4. OsVIL1 Represses OsCKX2 Expression

**Figure 6:**
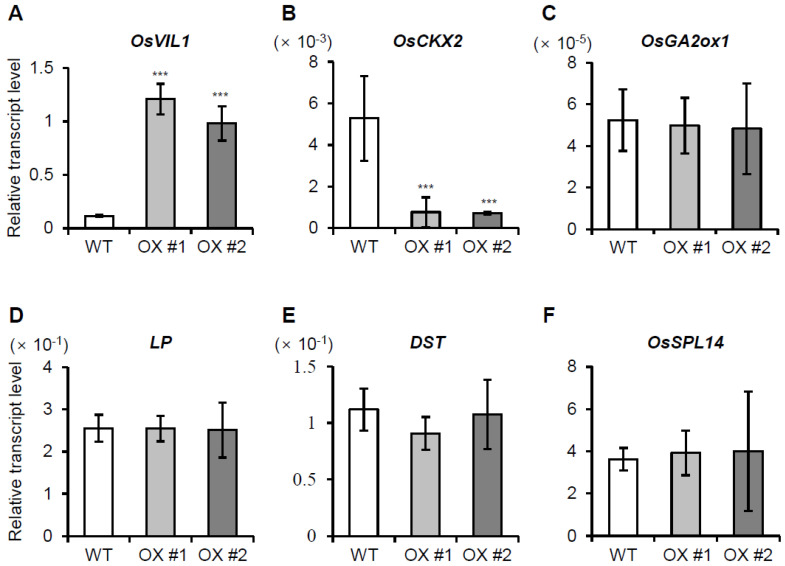
Expression levels of several genes regulating plant height and grain yield in *OsVIL1* overexpression (OX) plants. Samples were prepared from the cell division zones (0.5 cm) of the first internodes (4 cm) under paddy field conditions. The expression levels of *OsVIL1* (**A**), *OsCKX2* (**B**), *OsGA2ox1* (**C**), *OsLP1* (**D**), *OsDST* (**E**), and *OsSPL14* (**F**) in the first internode. Rice *Ubiquitin1* was used as an internal control. The error bars indicate the standard deviations, *n* = Statistical significance is indicated by *** (*p* < 0.001).

Previously, we showed that gene expression associated with cell division/cell cycle, cell organization, DNA synthesis, and protein synthesis/amino acid activation was increased in *OsVIL2* OX plants [7]. We also reported that OsVIL2 suppresses the expression of *OsCKX2*, which encodes a cytokinin-degrading enzyme [7]. The down-regulation of *OsCKX2* causes accumulation of active cytokinin that promotes cell division in *OsVIL2* OX plants [7]. Here, we investigated whether OsVIL1 also affected *OsCKX2* expression. Analyses of the samples from the cell division zones showed that *OsCKX2* expression was significantly reduced in OsVIL1 OX plants compared to WT (Figure 6B). However, the transcript levels of *GA 2-oxidase 1* (*GA2ox1*) that controls GA homeostasis by inactivating bioactive GA through 2β-hydroxylation [28] were not significantly altered (Figure 6C). Similarly, the expression levels of *LP*, *DST*, and *OsSPL14*, which regulate panicle size and grain yield [29,30], did not differ between the WT and *OsVIL1* OX plants (Figure 6D–F). Our results suggest that *OsVIL1* increases active cytokinin levels through the control of *OsCKX2* expression.

### 2.5. OsVIL1 Directly Suppresses OsCKX2 Expression by Regulating H3K27 Chromatin States

**Figure 7:**
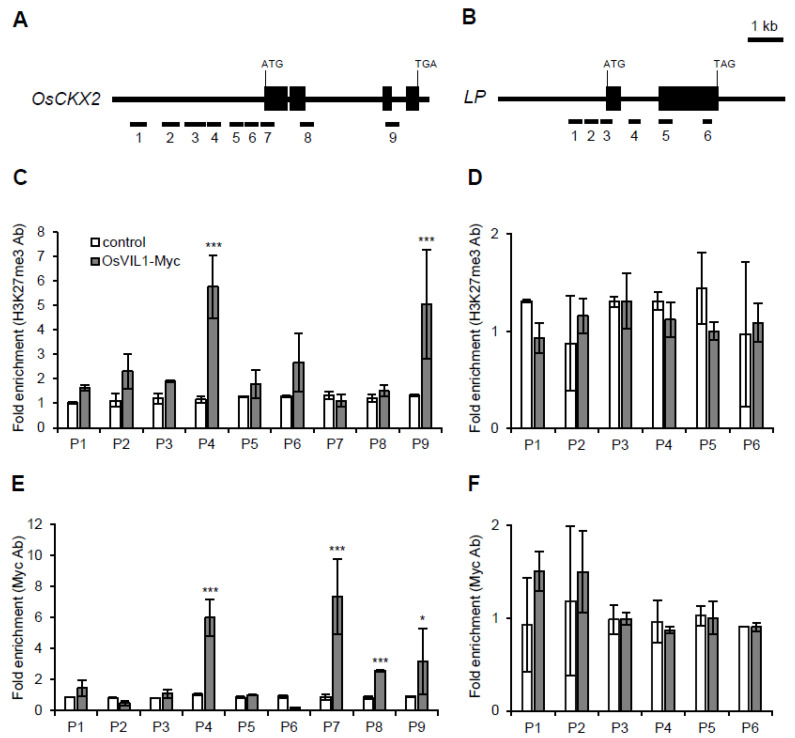
Chromatin immunoprecipitation assay of OsVIL1 in *OsCKX2* chromatin. Samples were prepared from the upper 0.5 cm of the cell division region in the second internode before the heading stage, *n* = 10. (**A**,**B**) Genomic structure of *OsCKX2* and *OsLP*. OsVIL1-Myc transgenic plants were used for the chromatin immunoprecipitation assay. As a control, we used Myc-empty transgenic plants. (**C**,**D**) Analysis of H3K27me3 chromatin states in *OsCKX2* chromatin (**C**) and *OsLP* (**D**). (**E**,**F**) Enrichment of *OsCKX2* (**E**) and *OsLP* (**F**) chromatin in OsVIL1-Myc transgenic plants. The entire experiment was conducted three times and the graph shown are from one of the three experiments. The error bars indicate the standard deviations. Statistical significance is indicated by *** (*p* < 0.001), * (*p* < 0.01).

OsVIL2 represses *OsCKX2* expression by regulating the chromatin states of H3K27me3 [7]. To investigate whether OsVIL1 also enhances H3K27me3 in *OsCKX2* chromatin, we generated transgenic plants that expressed Myc-tagged OsVIL1 as well as Myc alone as a negative control. To perform chromatin immunoprecipitation (ChIP) assay, the basal parts of the second internode were collected before the heading stage. When we observed the levels of H3K27me3 in *OsCKX2* chromatin using H3K27me3 antibodies (Figure 7A), the *OsCKX2* chromatin was significantly enriched in the OsVIL1-Myc transgenic plants compared with the transgenic plants expressing the Myc-tag alone (Figure 7C). Moreover, when we observed the direct binding of OsVIL1 in the *OsCKX2* chromatin region using anti-Myc antibodies, OsVIL1-Myc proteins were enriched at the *OsCKX2* chromatin sites P4 and P7–P9 (Figure 7E). As a negative control, we used *LP*, which was not significantly enriched in the OsVIL1-Myc transgenic plants (Figure 7D,F).

## 3. Discussion

### 3.1. OsVIL1 Is Involved in Chromatin Remodeling in Rice

The rice genome contains four *VIN3*-like genes (*OsVIL1–OsVIL4*) that are homologous to *Arabidopsis VIN3* [31]. They are composed of a PHD finger that is a conserved motif for histone binding, an FNIII domain, and a VID domain [20,21,25]. The PHD finger of OsVIL2 binds to the native histone H3 in vitro and induces H3K27me3 histone modification in the chromatin of target genes [7,23]. In this study, we showed that OsVIL1 also modifies chromatin states by regulating H3K27me3 to repress the expression of down-stream genes. H3K27me3 is an epigenetic modification of histone H3 and affects the stability and plasticity of gene regulation during various developmental processes. The chromatin interacting factor of OsVIL2 binds to OsEMF2b, which is a core protein in the PRC2 complex, and induces flowering by repressing *OsLFL1* in rice [23]. The *osvil2* null mutants display late flowering phenotypes, as do *OsEMF2b* mutants. Although *OsVIL2* OX plants display increased biomass and grain yield, the overexpression of *OsEMF2b* does not (unpublished data). Similar to OsVIL2, OsVIL1 represses the expression of target genes by inducing H3K27meMoreover, OsVIL1 binds to OsEMF2b through the FNIII domain [25]. However, further studies are needed to elucidate the molecular mechanisms that underlie how the interactions between the OsVIL proteins and OsEMF2b control biomass and grain yield in rice.

### 3.2. VIN3-like Proteins Regulate Cell Division of Meristem in Rice

Vernalization is an acceleration of floral transition from vegetative growth to reproductive growth after exposure to a prolonged period of low temperature [31,32,33]. In Arabidopsis, VIN3 that is a repressive chromatin-remodeling component is induced only after perceiving a sufficient duration of cold [34,35]. The VIN3 protein induces the trimethylation of H3K27 of the target loci to regulate gene expression. For example, VIN3 increases the levels of H3K27me3 in the *FLC* chromatin region and other *FLC* clade members [18]. Rice does not require vernalization to control flowering time, and no *FLC*-homologous gene is present in rice [23]. Although the detailed mechanisms for controlling flowering time differ between rice and Arabidopsis, the OsVIL1 and OsVIL2 proteins in rice play a role in promoting flowering by reducing the expression of *OsLFL1*, a flowering repressor [23,25].

In rice, *osvil2* null mutants display pleiotropic phenotypes, including altered flowering times, reduced tiller numbers, alterations in the leaf angles, and defects in floral organ development [23,26,27]. In addition, *OsVIL2* OX plants exhibit increased biomass and grain yields caused by an accumulation of active cytokinin [7]. In this study, we showed that overexpression of *OsVIL1* led to similar phenotypes as those seen in *OsVIL2* OX plants, including increased biomass and grain yield. When we examined the histone modification states of *OsCKX2* chromatin, a gene responsible for cytokinin degradation, OsVIL1 affected trimethylation of H3K27 in *OsCKX2* chromatin, similarly to OsVIL2. Cytokinin plays an essential role in cell proliferation and regulating the size of meristem [4]. In rice, the VIN3-like proteins OsVIL1 and OsVIL2 repressed *OsCKX2* expression to create a balance between cytokinin synthesis and catabolism to control meristem activity and cell proliferation. In light of these results, it will be interesting to study how VIN3-like proteins in other plant species differ from those in rice and Arabidopsis.

### 3.3. Loss-of-Function of OsVIL1 Causes a Reduction in Fertility

Although similar phenotypes were seen when *OsVIL1* or *OsVIL2* were overexpressed, the *osvil1* null mutants did not show remarkable developmental phenotypes in contrast with *osvil2* null mutants, which exhibit pleiotropic phenotypes. Because both OsVIL1 and OsVIL2 induced the trimethylation of H3K27 in the *OsCKX2* chromatin region, we expected that their PHD domains would share similar conserved target loci. In addition, OsVIL1 and OsVIL2 bind to OsEMF2b, the core protein of the PRC2 complex, through the FNIII domain. *OsEMF2b* null mutants display abnormal floral organ identity, reduced tiller size, and pleiotropic phenotypes as in *osvil2* null mutants [23]. For these reasons, we suggested that OsVIL2 functions with OsEMF2b in the PRC2 complex to regulate cell division and meristem identity. The null mutants in *OsVIL1* did not cause observable developmental defects except in fertilization, suggesting that OsVIL2 can rescue most of OsVIL1 functions. The cause of the reduced fertility of the *osvil1* mutant has not yet been clearly identified. The *osvil2* has no research results on pollen or the anther development because the floral organ is completely broken in this mutant. The OsEMF2b, interaction partner of OsVIL1 and OsVIL2, functions in anther and pollen development in rice [36]. Both OsVIL1 and OsVIL2 are expected to plays a role in anther and pollen development, but further studies are needed to identify more detailed functions.

However, biomass and grain yield increased when OsVIL1 was overexpressed, indicating that OsVIL1 serves to assist the functions of OsVIL2 in regulating cell division and meristem activity. Further study, such as generation of double, triple, or quadruple mutants of OsVIL gene mutants, is required to reveal detailed molecular mechanisms of interaction among the four OsVIL proteins to determine how they regulate various developmental processes.

## 4. Materials and Methods

### 4.1. Plant Materials and Growth Conditions

*Oryza sativa* var. *japonica* cultivar Nipponbare was used to generate transgenic plants. Target regions for the CRISPR/Cas9-induced *osvil1* KO mutants were genotyped through sequencing (Table S1). For DNA sequencing of the target regions, PCR products were gel purified using *Pfu* polymerase and subjected to subcloning. More than 10 transformed *E.coli* colonies for each line were then randomly picked up and sequenced. DNA mutations were subsequently identified through sequence alignment between sequenced alleles and WT alleles using NCBI BLAST (https://blast.ncbi.nlm.nih.gov (accessed on 1 November 2021). Seeds were germinated either on an MS medium or in soil. We selected two T_0_ lines that highly expressed the introduced *OsVIL1* and renamed the recombinant gene OX #1 and #The T_1_ progeny were grown on an MS medium containing 40 μg mL^−1^ hygromycin. Finally, plants were grown either in the paddy field or a controlled growth room under a light/dark cycle (14.5-h light at 28 °C/10-h dark at 23 °C; 50% humidity) as previously reported [37].

### 4.2. Vector Construction and Rice Transformation

To generate *OsVIL1* OX plants and Myc-tagged transgenic plants, *OsVIL1* full-length complementary DNA (cDNA) was amplified and digested with restriction enzymes *Spe*I and *Sac*I, and inserted into binary vector pGA3426 that contains the maize *Ubiquitin 1* (*Ubi1*) promoter, or pGA3438 that contains the maize *Ubiquitin1* (*Ubi1*) promoter and Myc epitope [38]. To construct the CRISPR/Cas9 plasmid, the target sequence was cloned into the entry vector pOs-sgRNA; then, the target sequence and sgRNA were further cloned into a destination vector, pH-Ubi-cas9-7, using the Gateway^TM^ system as previously reported [39,40]. The constructs were introduced into *Agrobacterium tumefaciens* LBA4404 using the freeze-thaw method [41]. Rice transformation via Agrobacterium-mediated co-cultivation was performed as previously described [42]. All primers used for cloning are listed in Table S1.

### 4.3. RNA Extraction and Real-Time PCR Analysis

For RNA extraction, 0.5-cm samples were collected from the leaf blades or basal parts of the first and second internodes. Total RNA was isolated with RNAiso Plus (Takara, Kyoto, Japan) and 2 µg of total RNA were annealed with 10 ng of oligo (dT). First-strand cDNA was synthesized with Moloney murine leukemia virus reverse transcriptase (Promega, Fitchburg, WI, USA), RNasin (Promega, Fitchburg, WI, USA), and 2.5 mM deoxyribonucleotide triphosphate. Transcript levels were analyzed by quantitative RT-PCR (qRT-PCR) with SYBR Green I Prime Q-Master mix (GENETBIO, Daejeon, Korea) in a Rotor-Gene Q system (Qiagen, Hilden, Germany) [39]. For normalization, we used rice gene *Ubiquitin1* (*OsUbi1*) or *Actin1* (*OsActin1*) as an internal control. The relative expression level was calculated using the ΔΔCt method. All primers used for qRT-PCR are listed in Table S1.

### 4.4. Histochemical Analysis

To observe internode development, we used paraffin to obtain sections from the samples of the first internode. The samples were collected from the basal and middle parts of the first internode, and fixed with FAA solution as previously reported [43]. Then, samples were dehydrated and infiltrated with paraffin before being embedded in the embedding ring. Samples were cut to a thickness of 10 µm, rehydrated, and stained with toluidine blue to observe cell differentiation and elongation. Samples were visualized using a BX61 microscope (Olympus, Tokyo, Japan).

### 4.5. Chromatin Immunoprecipitation (ChIP) Analysis

Transgenic plants expressing OsVIL1-Myc were used for ChIP analysis as previously reported [44]. Briefly, about 2 g of the basal parts from the second internodes were collected and incubated in 3% formaldehyde; then, the nuclei were isolated. Chromatins were fragmented to approximately 500 to 1000 bp lengths by sonication. Before preclearing, 1% of the sample was gathered as an input. For immunoprecipitation, we used anti-Myc monoclonal antibodies (#2276; Cell Signaling, Bethesda, MD, USA) as described previously [23,39]. Data were normalized according to the percentage input method as reported previously [45]. The primer sequences used for the ChIP assays are listed in Table S1. All experiments were conducted at least three times, each involving three biological replicates.

### 4.6. Statistical Analyses

Using R software, the *p* values were obtained by using one-way analysis of variance (ANOVA; Tukey’s HSD test) for the test groups [46].

### 4.7. Phylogenetic Analysis

The orthologous and/or closely paralogous *OsVIL1* genes among the 44 representative plants were identified, using an OrthoFinder pipeline (version 2.5.1) [47]. Afterward, phylogenetic relationships among these VIL genes were retrieved as previously reported [48]. Briefly, multiple alignment of the coding sequences from identified VIL genes were aligned using MAFFT (version 7.243) [49]. Next, the Neighbor-Joining (NJ) phylogenetic tree was inferred through MEGA6 [50] with bootstraps = 1000.

### 4.8. Meta-Expression Analysis

To analyze tissue specific expression patterns of OsVIL genes, we used a CAFRI-Rice database site (http://cafri-rice.khu.ac.kr/inspector (accessed on 20 December 2021) [51] based on public available rice Affymetrix microarray data set from the NCBI Gene Expression Omnibus (GEO). The expression values are normalized by R language and then turned into log2 values. With downloaded log2 expression values, we constructed the heatmap with TB tools program [52].

## Data Availability

All data is comprised in the manuscript.

## Supplementary Materials

The following are available online at https://www.mdpi.com/article/10.3390/plants11010083/s1, Figure S1. Expression pattern heatmaps of OsVIL genes; Figure S2. Longitudinal section of the first internode; Table S1. Primers used for this study.
