# Supplementary material for: A VIN3-like Protein OsVIL1 Is Involved in Grain Yield and Biomass in Rice
Source: Plants (Basel). 2021 Dec 28;11(1):83. doi: 10.3390/plants11010083 (PMC8747509; doi:10.3390/plants11010083)
Supplement: Supplementary file 1 [file plants-11-00083-s001.zip › plants-1504177-supplementary.pdf]

plants

Supplementary Material

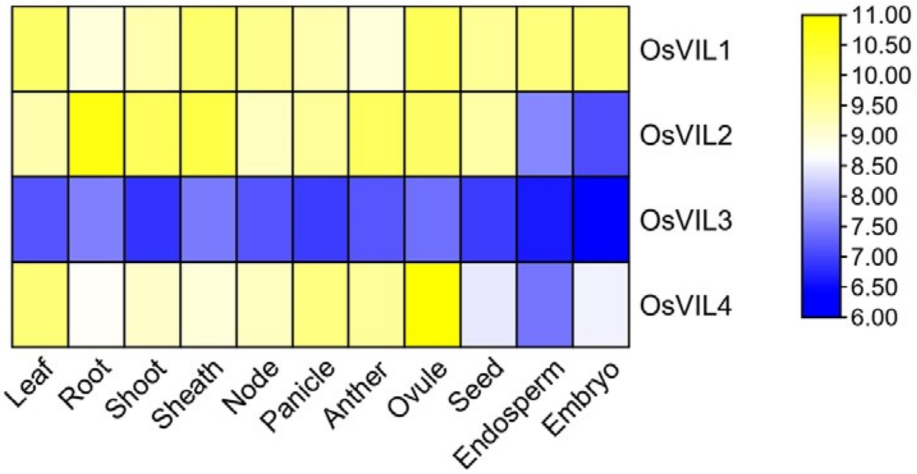

Figure S1. Expression pattern heatmaps of OsVIL genes.

.

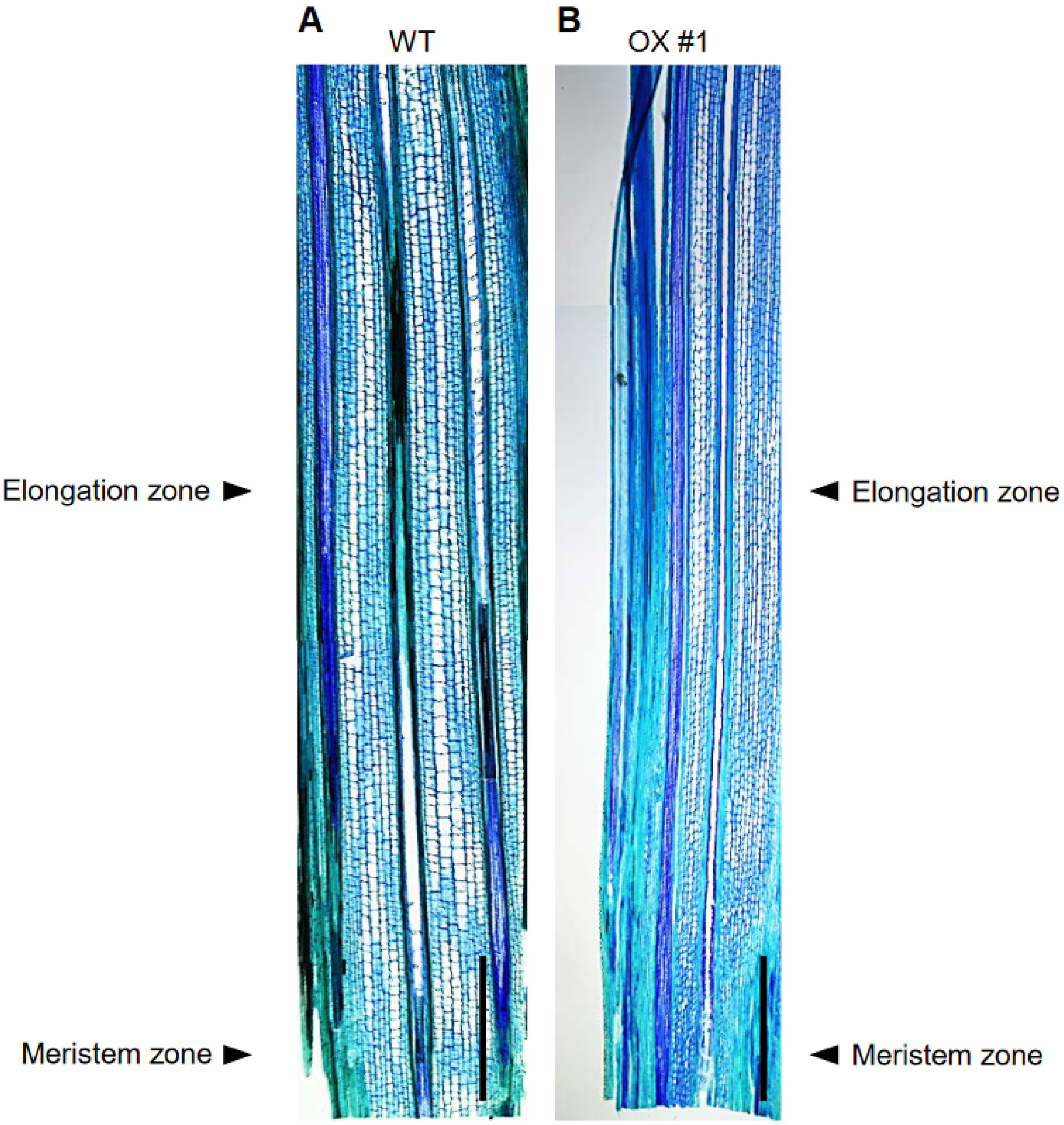

Figure S2. Longitudinal section of the first internode. **(A)** The first internode from wild-type (WT). **(B)** The first internode from *OsVIL1* overexpression (OX) #1. Bars = 1 mm.

Table S1. Primers used for this study.

Name	Sequence (5'-3')	Purpose
qRT-Ubi1-F	AACCAGCTGAGGCCCAAGA	RT-PCR
qRT-Ubi1-R	ACGATTGATTTAACCAGTCCATGA	
qRT-OsActin1-F	CCTCTTCCAGCCTTCCTTCAT	
qRT-OsActin1-R	ACGGCGATAACAGCTCCTCTT	
qRT-OsCKX2-F	TGTCCCTTCTACAATGGTGC	
qRT-OsCKX2-R	CATCCTGACCTGCTCTTGCT	
qRT-OsGA2ox1-F	TCAATGTTGGTGATGTCCTC	
qRT-OsGA2ox1-R	GCTGGCTGTGATTGTCTCT	
qRT-DST-F	GCTACTGCTGGCGTTGGG	
qRT-DST-R	GAGATGGTGCTGGTGCGT	
qRT-OsLP-F	GTGAAGAGATGATGTCATTG	
qRT-OsLP-R	GGCATCATCCCTCCCTCTAT	

qRT-OsSPL14-F	CAAGGGTTCCAAGCAGCGTAA	
qRT-OsSPL14-R	TGCACCTCATCAAGTGAGAC	
OsVIL1_OX_BsiWI_F	CGTACGCTTCTGCTGCTGCTGGGATG	Transgenic plants
OsVIL1_OX_SpeI_R	ACTAGTTTAGTGCCATAACTTACTGC	
OsVIL1_CR_F	GGCAAGATAATACCCTGGAACAGT	
OsVIL1_CR_R	AAACACTGTTCCAGGGTATTATCT	
OsVIL1_CR_geno_PCR_F	CATGCCTAGGTTTTGGCACTAC	Genotyping
OsVIL1_CR_geno_PCR_R	AGACCAGCCAAAGGCTAGGAT	
OsCKX2-1F	CACCTTGTCCCTTCTACAAT	Chromatin Immunoprecipitation
OsCKX2-1R	GACGAAGCAGTTGAGCATGA	
OsCKX2-2F	AACTCCCTTGTGGAGTACAA	
OsCKX2-2R	TCTGTGTGACAAGGACTGAA	
OsCKX2-3F	AGGTGAACTAATTGGCTGAA	
OsCKX2-3R	GGCCGGTAACACATTTGAAC	
OsCKX2-4F	AGGTGAACTAATTGGCTGAA	
OsCKX2-4R	GACCACACATCCAAAAATAAA	
OsCKX2-5F	GGACTCGAGCAAGGTACCAA	
OsCKX2-5R	AACACATCCTTTTTGAGACG	
OsCKX2-6F	GATATGGGGACGTCGTGACT	
OsCKX2-6R	CTTCTCGTTCACCCTGAAGTG	
OsCKX2-7F	AAAAAGATCCGTGCATTTCAA	
OsCKX2-7R	TCCTAGATGGACCGAGGAAA	
OsCKX2-8F	ATGATGGCGGGAACAACTAA	
OsCKX2-8R	CTTAATTGCATGCGTGCGTA	
OsCKX2-9F	CATGGCTGAACCTGTTCCT	
OsCKX2-9R	ATGAGGGGTCGTCATTTTGA	
OsLP-1F	CGTTGCGACTTTCATTATATGC	
OsLP-1R	GGACTAAGTCGAGACTTCTAACGA	
OsLP-2F	AACCTTTCTACTCGTTAGAAGTCTCG	
OsLP-2R	CATTCAACATATTACTGTATTGGAAAA	
OsLP-3F	TGAGCATAAGAAGCTGAAAAGAAA	
OsLP-3R	GAGGGAGCTGAGAGGGAAAG	
OsLP-4F	TTGGTTCTTTCTTCCCTCCA	
OsLP-4R	TCTTGGCGGGTTCACTAATC	
OsLP-5F	TACTGTGCTGTTCGCTGGAT	
OsLP-5R	AAAAGATCATCAGGGAGAACG	
OsLP-6F OsLP-6R	GATTTGGCGAGTTCGATGAT TGCAGAG-GAAACCTCTTCGT	
